# “Postnatal” Outpatient Program for Postpartum Women at the University Psychiatric Hospital Vrapce, Zagreb

**DOI:** 10.1192/j.eurpsy.2025.877

**Published:** 2025-08-26

**Authors:** S. Martic-Biocina

**Affiliations:** 1 Department of Social Psychiatry, Psychiatric Clinic Vrapce, Zagreb, Croatia

## Abstract

**Introduction:**

Postpartum mental health challenges are common, with some women experiencing anxiety, depressive symptoms, or difficulties related to childcare and familial support. To address these issues, the University Psychiatric Hospital Vrapče in Zagreb, Croatia, launched the “Postnatal” outpatient program in 2023, offering structured support for women in the postpartum period.

**Objectives:**

The objective of the program is to provide psychological support for women with children aged 0–2 years through a combination of online and in-person group therapy, to address postpartum mental health issues and provide a supportive environment.

**Methods:**

The program is promoted through the clinic’s website, social media, maternity hospitals, pediatric centers, and visiting nurse services. Women join the program through self-referral or on the recommendation of healthcare professionals. A team of three psychiatrists conduct an initial assessment, and approximately 80% of those evaluated are admitted into the program. The program comprises two 90-minute weekly sessions: an online educational workshop and an in-person group therapy session. Each cycle lasts for 8 weeks, followed by monthly 90-minute in-person group meetings.

**Results:**

A total of 45 participants have completed the program so far. About 30% of participants met the clinical criteria for anxiety or depressive disorders, while there were two cases of postpartum psychosis. The majority reported non-specific concerns, such as tension, insomnia, fatigue, and emotional instability. Around 30% indicated a lack of support from their partners, and 10% had pre-existing psychological diagnoses (including OCD, borderline personality disorder, and anxiety-depressive disorder). Most participants were first-time mothers, with around 10% being second or third-time mothers. In the program, participants share experiences related to conception, pregnancy, maternity hospital stays, postpartum mental health changes, and the challenges of maternal care. Participation rates have been consistently high, with only one dropout. Post-program evaluations show a high level of satisfaction, with participants highlighting the group meetings and peer support as the most valuable aspects.

**Conclusions:**

The “Postnatal” program has demonstrated positive outcomes in providing support to postpartum women. The combination of educational workshops and group therapy has been effective in addressing both clinical and non-specific postpartum challenges. Further cycles and long-term follow-ups are recommended to evaluate the program’s sustained impact.

**Disclosure of Interest:**

None Declared

